# Breast Cancer Secondary to Radiation Therapy in a Patient With Wilms Tumor

**DOI:** 10.7759/cureus.23597

**Published:** 2022-03-29

**Authors:** Lintu Ramachandran, Ghanshyam Patel, Safa Fatima, Mohammad Khan

**Affiliations:** 1 Internal Medicine, Javon Bea Hospital, Rockford, USA

**Keywords:** breast cancer, radiation associated cancer, cancer screening, wilms tumor, secondary malignancy

## Abstract

Wilms tumor, one of the most common childhood malignancies, is typically treated with a combination of chemotherapy, radiation, and surgical resection. Wilms tumor survivors who received radiation therapy are, however, at a higher risk of secondary malignancies and need vigilant monitoring. We present the case of a 35-year-old female with history of Wilms tumor at age five, who received radiation therapy for pulmonary metastasis, and was found to have breast cancer at the age of 35. We discuss different protocols in treatment of Wilms tumor and current secondary malignancy screening recommendations. We also recognize the importance of screening guideline awareness among primary care physicians and its mortality and morbidity implications.

## Introduction

Wilms tumor (WT) is the most common childhood renal malignancy and is often diagnosed before the age of five. The estimated incidence of WT is one in 10000 infants [1]. Children with Beckwith-Wiedemann syndrome; Wilms tumor, aniridia, genitourinary abnormalities and range of developmental delays (WAGR) syndrome; and Denys-Drash syndrome are up to 50% risk of developing WT and are usually screened every four months until the age 7 with renal ultrasonography [2]. The common sites of metastases for WT are the lung and liver. In instances of patients who have pulmonary metastasis, the standard of care includes chest radiation therapy [3]. The female survivors of childhood invasive cancer who are treated with radiation therapy including chest, are at a high risk of developing secondary breast malignancy [4]. We report a patient with WT who received radiation, who did not undergo any screening and subsequently developed breast cancer.

## Case presentation

**Figure 1:**
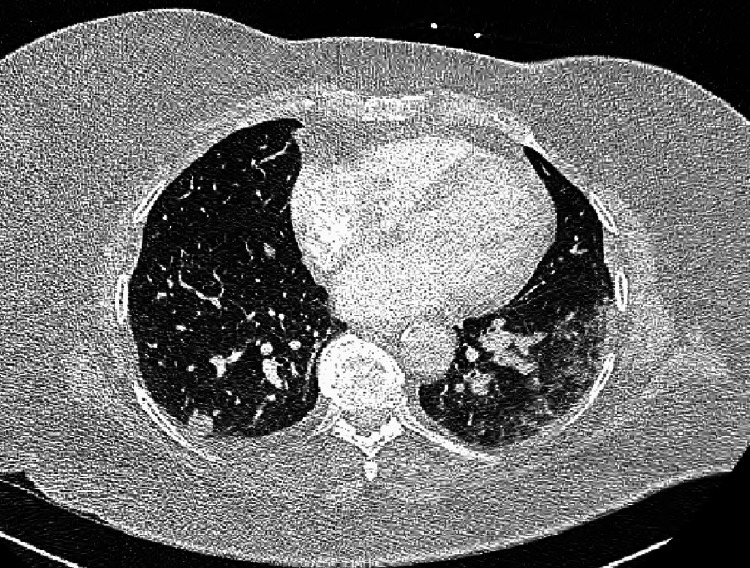
CT of the chest with contrast showing multiple bilateral pulmonary nodules

The patient is a 35-year-old female with a past medical history of bicuspid aortic valve status post aortic valve replacement, hypothyroidism, and WT with pulmonary metastasis status post left nephrectomy, chemotherapy and whole chest radiation therapy at age five, who presented to the outpatient clinic with a breast mass noted on self-examination for six weeks. The exact chemotherapy regimen and the dosage of radiation were unknown and lost to paper charts. The mass was described as a painless fixed mass that did not change in size based on her menstrual cycle. She denied any galactorrhea, weight changes, cold or heat intolerance, dysmenorrhea, menometrorrhagia or any other new onset issues. Her family history was negative for any malignancy including breast cancer. Her surgical history was remarkable for aortic valve replacement and left nephrectomy. She denied any tobacco abuse, alcohol use or recreational drug use. Vitals and laboratory results including a comprehensive metabolic panel, complete blood count, prolactin levels, and TSH were normal. A physical exam showed a 2cm x 2cm palpable mass in the left outer quadrant of the left breast. Mammography showed an irregular mass in the lateral left breast and a BI-RADS classification of class V. Ultrasound confirmed the solid mass, and a subsequent biopsy showed triple-negative invasive ductal carcinoma. The patient underwent a bilateral nipple-sparing mastectomy and a right axillary sentinel lymph node biopsy that showed advanced breast cancer. A CT of the chest/abdomen, pelvis and an MRI of the brain did not show any suspicious malignancies. Despite undergoing chemotherapy, radiation therapy and reconstructive surgeries for her breast cancer, six months later the patient developed axillary lymphadenopathy, shortness of breath and speech difficulty. A CT of the chest with contrast revealed bilateral pulmonary nodules as noted in Figure 1.

**Figure 2:**
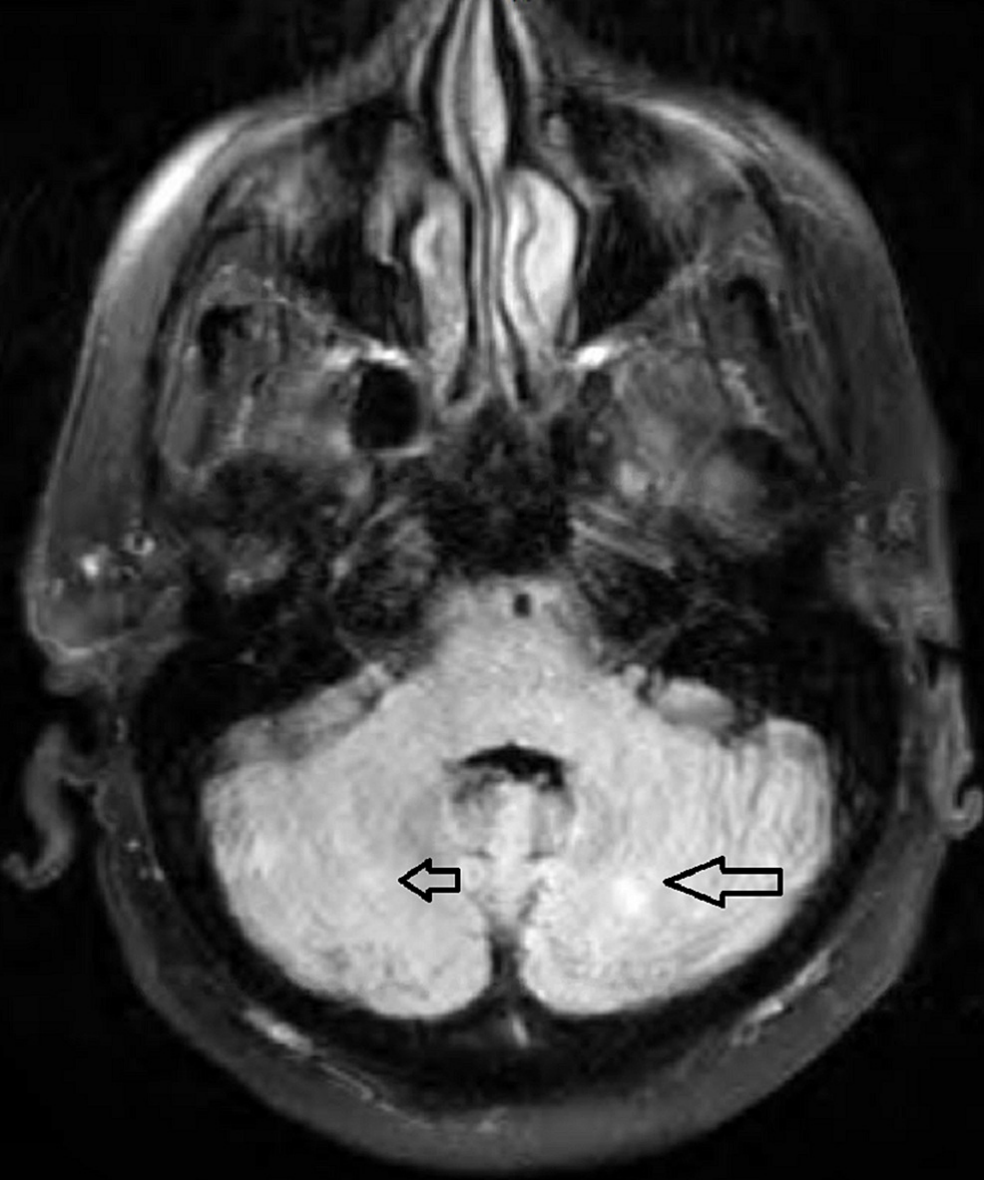
T2 flair sequence of brain MRI with and without contrast showing multiple metastatic lesions in the cerebellum (black arrows)

**Figure 3:**
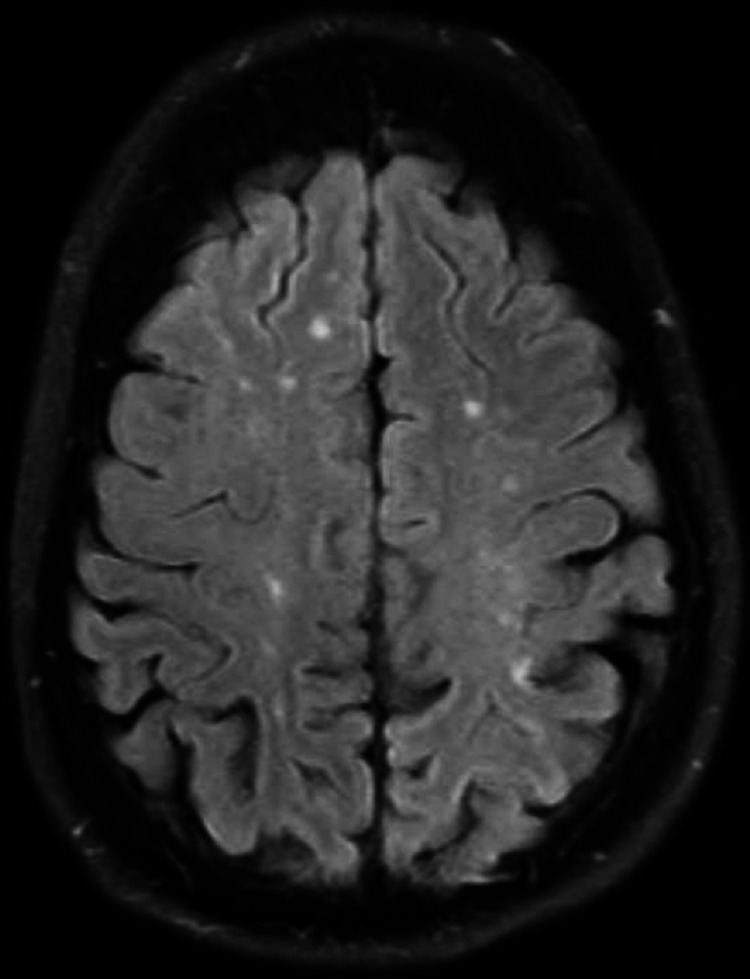
T2 sequence of brain MRI with contrast showing bilateral cerebral hemispheres

The results of the MRI revealed multiple lesions in the bilateral cerebellum (Figure 2) and cerebral hemispheres (Figure 3).

The patient's status quickly continued to deteriorate. She elected comfort care and passed away.

## Discussion

Wilms tumor is the most common childhood renal malignancy and accounts for roughly 87% of all pediatric renal malignancies [5]. Approximately 650 new cases of WT are reported in the United States annually [6]. Treatment of Wilms tumor is often multimodal, involving surgery, chemotherapy and/or radiotherapy. Pulmonary radiotherapy used to be recommended early in the course of disease for all patients with WT who had evidence of pulmonary metastases on X-ray or chest CT [7]. Due to advancements in chemotherapy, radiation therapy and molecular biology, currently, radiation therapy recommendation is based on staging and response to chemotherapy [8]. Our patient received whole lung radiation therapy after being found to have pulmonary metastasis on a CT of the chest. 

Two protocols exist currently for the treatment of WT - The National Wilms Tumor Study/ Children's Oncology Group (NWTS/COG) and the International Society of Pediatric Oncology (SIOP) guidelines. The NWTS/COG protocol dictates that full nephrectomy be performed first to assess the histology and staging. The staging can then determine further course of action [9]. The SIOP protocol, on the other hand, dictates that patients get chemotherapy prior to the surgery to shrink the tumor prior to resection [10]. Both interventions have high rates of overall survival. While the NWTS/COG approach prevents unnecessary chemotherapy for benign tumors, the SIOP diminishes tumor spillage and makes surgical resection easier [11]. Our patient had complete unilateral left nephrectomy first and then underwent chemotherapy and radiation therapy as per the NWTS/COG guidelines.

Another significant difference between the two protocols is the need for radiation therapy. The NWTS/COG guidelines recommend radiation therapy to the tumor bed for all patients with stage III WT [12]. The SIOP guidelines only recommended pulmonary radiotherapy if there is no complete response after week 10 post-surgery [13]. Since both treatment protocols include chemotherapy and radiation, several cases have reported a high risk of developing secondary malignancies. The estimated incidence of a secondary malignancy after WT is 0.6% at 10 years, 1.6% at 20 years, and 3.8% at 30 years [14]. Survivors of WT who received radiation therapy also seem to be at higher risk of developing secondary malignancy [15]. A prospective British childhood cancer survivors’ study of WT survivors attributed 50% of deaths to secondary neoplasms and 25 % of deaths to cardiac-related causes, and radio therapy was a risk factor for both outcomes. Bowel and breast cancers were the most frequent subsequent neoplasms [16]. Our patient developed breast cancer and eventually passed away from the breast cancer at age 36.

Among childhood cancer survivors, the cumulative breast cancer incidence ranges from 13% to 20% by age 40 to 45 years [17]. The latency period of breast cancer after chest irradiation ranges from eight to 10 years, and the risk increases linearly with radiation dose [18]. Current, guidelines recommend yearly mammography and breast MRI starting at age 25 or eight years after treatment culmination, only for those who received ≥20Gy chest or mantle radiation. Mantle radiation has been historically used in the treatment of Hodgkin's lymphoma [19]. Screening with mammography and MRI is projected to avert half of the expected breast cancer deaths among high-risk survivors [20]. Our patient developed breast cancer that was detected late and in an advanced stage with her major risk factor for cancer being the radiation she received for her WT at age five. Neither the patient nor the family recalled being talked to about the importance of long-term follow-up after treatment. Our patient’s last known imaging was a CT chest/abdomen/pelvis that was done at age 10, and she did not have screening mammograms despite having seen a primary care physician regularly since age 18.

## Conclusions

Our case demonstrates the importance of early screening for WT survivors. Female WT survivors treated with chest radiation therapy are at high risk of early breast cancer and need close monitoring. Physicians, especially primary care physicians should be cognizant of the importance of the cancer screening guidelines as early detection may have lifesaving implications. In our patient’s case, screening per guidelines could have significantly altered her prognosis and quality of life.
